# N-terminal Domain of Prion Protein Directs Its Oligomeric Association*

**DOI:** 10.1074/jbc.M114.566588

**Published:** 2014-07-29

**Authors:** Clare R. Trevitt, Laszlo L. P. Hosszu, Mark Batchelor, Silvia Panico, Cassandra Terry, Andrew J. Nicoll, Emmanuel Risse, William A. Taylor, Malin K. Sandberg, Huda Al-Doujaily, Jacqueline M. Linehan, Helen R. Saibil, David J. Scott, John Collinge, Jonathan P. Waltho, Anthony R. Clarke

**Affiliations:** From the ‡ Department of Neurodegenerative Disease, MRC Prion Unit, UCL Institute of Neurology, Queen Square, London WC1N 3BG,; the § Institute of Structural and Molecular Biology, Birkbeck College, Malet Street, London WC1E 7HX,; the ¶ National Centre for Macromolecular Hydrodynamics, School of Biosciences, University of Nottingham, Sutton Bonington Campus, Leicestershire, LE12 5RD,; the ‖ ISIS Spallation Neutron and Muon Source and; ** Research Complex at Harwell, Rutherford Appleton Laboratory, Oxfordshire, OX11 0FA, and; the ‡‡ Department of Molecular Biology and Biotechnology, Krebs Institute for Biomolecular Research, University of Sheffield, Sheffield S10 2TN, United Kingdom

**Keywords:** Amyloid, Intrinsically Disordered Protein, Prion, Protein Aggregation, Protein Folding, β-PrP, β-Sheet, CJD, Molten Globule, Oligomer

## Abstract

The self-association of prion protein (PrP) is a critical step in the pathology of prion diseases. It is increasingly recognized that small non-fibrillar β-sheet-rich oligomers of PrP may be of crucial importance in the prion disease process. Here, we characterize the structure of a well defined β-sheet-rich oligomer, containing ∼12 PrP molecules, and often enclosing a central cavity, formed using full-length recombinant PrP. The N-terminal region of prion protein (residues 23–90) is required for the formation of this distinct oligomer; a truncated form comprising residues 91–231 forms a broad distribution of aggregated species. No infectivity or toxicity was found using cell and animal model systems. This study demonstrates that examination of the full repertoire of conformers and assembly states that can be accessed by PrP under specific experimental conditions should ideally be done using the full-length protein.

## Teaser

**Background:** Non-fibrillar oligomers are implicated as neurotoxic species in several amyloid neurodegenerative diseases.

**Results:** Full-length prion protein (PrP) forms distinct non-fibrillar β-sheet-rich oligomers. Truncated protein, lacking the N terminus forms nonspecific aggregates.

**Conclusion:** The unstructured N terminus of PrP is key to the folding and aggregation of its structured region.

**Significance:** To examine the full repertoire of PrP conformers and assembly states, full-length protein should be used.

## Introduction

Prion diseases are a group of fatal neurodegenerative diseases that include bovine spongiform encephalopathy in cattle, scrapie in sheep and goats, and Creutzfeldt-Jakob disease, Gerstmann-Sträussler-Scheinker disease, fatal familial insomnia, and kuru in humans (1).

According to the “protein-only” hypothesis, the key molecular event in the pathogenesis of these diseases is the conversion of the host-encoded prion protein (PrP)^8^ from its normal cellular conformation (PrP^C^) to disease-associated isoforms often referred to as PrP^Sc^, which may form amyloid deposits. Although the precise molecular events involved in this conversion and the precise structure of infectious prions remain ill-defined, molecular genetic and *in vitro* studies suggest that PrP^Sc^ acts as a template that promotes the conversion of PrP^C^ to PrP^Sc^ and that the difference between the two isoforms lies purely in the monomer conformation and its state of aggregation (1, 2).

PrP^Sc^ is classically defined in terms of its detergent insolubility and relative protease resistance and has a high β-sheet content (3–5). In contrast, PrP^C^ consists of a predominantly α-helical structured domain and an N-terminal segment, which is unstructured in solution conditions, with a single disulfide bond forming an integral part of the core of the C-terminal structured domain (6–8).

Although it is clear that prion pathology is generally associated with PrP aggregation and the deposition of abnormal protein deposits, it is increasingly recognized that there are multiple disease-related forms of PrP, including protease-sensitive species, which might comprise the majority of infectivity in some isolates (9–11). In particular, small non-fibrillar β-sheet-rich oligomers have been suggested to be the most efficient mediators of prion infectivity (12), and have been shown to exhibit more neurotoxicity both *in vitro* and *in vivo* than the fibrillar forms of PrP^Sc^ (13). Small oligomeric species have also been implicated in other amyloid-related diseases (14–20) and may provide targets for diagnostic and therapeutic treatment.

Several non-fibrillar oligomers have been obtained through the *in vitro* misfolding of PrP (13, 21–31), ranging in size (10–50 nm diameter) and in the minimum number of monomer subunits required (8–10 monomers) to form the oligomer. Generally these oligomers are rather aggregation prone and appear as transient species during the conversion to larger fibrils. The transient nature of these small oligomers has made it difficult to study their properties, structure, and relationship to fibrils and their physiological role (16). Whether such oligomers represent on- or off-pathway intermediates to amyloid formation remains contentious, however many of them display increased β-sheet structure and resistance to proteolysis, despite being soluble under physiological conditions (13, 22, 23, 26, 32, 33).

One such PrP species, termed β-PrP, is formed when PrP is refolded at acidic pH in a reduced state, with the disulfide bond broken (28, 34). This form of the protein assembles into soluble oligomers that have significant β-sheet content and partial resistance to proteinase K digestion, both properties characteristic of PrP^Sc^, and also forms amyloid fibrils which closely resemble those isolated from diseased brains (34, 35). In addition, β-PrP is antigenically distinct from native PrP^C^ and inhibits proteasome activity at nanomolar concentrations, a mechanism by which PrP^Sc^ has been proposed to effect neuronal death (36–38). Preincubation of β-PrP with an antibody specific for oligomerized proteins relieves this inhibition, consistent with oligomeric species mediating this effect (37). These data suggest a mechanism for intracellular toxicity mediated by defined oligomers of misfolded prion protein. β-PrP-associated inhibition of proteasome activity is most potent when full-length PrP (residues 23–231) is refolded to the β-PrP conformation (β-PrP^23–231^), rather than the shortened PrP molecule comprising residues 91–231 (β-PrP^91–231^). This suggests that PrP is capable of adopting distinct conformational isoforms that are dependent on the length of its polypeptide chain. To resolve this and also to clarify its relationship to PrP^Sc^, we have characterized the physical and biological properties of full-length β-PrP^23–231^, in particular its structural organization and potential toxicity and infectivity. We demonstrate that the intrinsically unstructured N-terminal region of the protein is of crucial importance in the intermolecular association and folding of the disulfide-reduced form of prion protein.

## EXPERIMENTAL PROCEDURES

### 

#### 

##### Protein Expression, Purification, and Preparation

Recombinant mouse PrP proteins were prepared and purified as previously described, including the preparation of labeled (^15^N and ^13^C/^15^N) protein for NMR studies (39). The purification of full-length PrP^23–231^ (PrP residues 23–231) differed slightly from that of PrP^91–231^ (PrP residues 91–231) in that following the initial nickel-nitriloacetic acid purification step and cleavage of the His tag, the final purification step involved reapplication of the protein to a second nickel-nitriloacetic acid column equilibrated in 20 mm BisTris, 25 mm imidazole, pH 6.5, and elution with 1 m imidazole in the same buffer.

Protein samples were converted from the normal α-helical conformation (α-PrP) to the β-sheet conformation (β-PrP), by reducing the protein disulfide bond and refolding at acidic pH as previously described (28, 34). Briefly, this was performed by denaturation of PrP in 6 m GdnHCl in the presence of 100 mm DTT to a final concentration of no more than 1 mg/ml, and subsequent refolding by dialysis against 10 mm sodium acetate, 2 mm DTT, pH 4. β-PrP^91–231^ samples were subject to ultracentrifugation at 150,000 × *g* for 4 h. β-PrP^23–231^ samples were not subjected to a similar ultracentrifugation step after establishing that the yield, CD spectrum, and sedimentation coefficient distribution of the β-PrP^23–231^ preparations were not affected by the centrifugation procedure. β-PrP^23–231^ was assembled into fibrils by treating 0.27 mg/ml of β-PrP^23–231^ in 10 mm sodium acetate, pH 4.0, with 1/9 volume of a 5 m stock of GdnHCl or NaCl (in the same buffer) to give final protein and denaturant concentrations of 0.25 mg/ml and 0.5 m, respectively.

##### CD Spectroscopy

Far UV CD spectra were acquired on a JASCO J-715 spectropolarimeter using typically 0.1-mm path length cuvettes and 10 accumulations, 1 nm bandwidth, 1 s^−1^ integration. Near UV CD spectra were typically acquired using 2-mm path length cuvettes and 30 accumulations. β-PrP spectra were acquired in 10 mm sodium acetate, 2 mm DTT, pH 4, α-PrP in 20 mm BisTris, pH 6.5. Mean residue ellipticity was calculated as [θ]R deg cm^2^ dmol^−1^ res^−1^.

##### NMR Spectroscopy

NMR spectra were acquired at 298 K on Bruker DRX-600 and DRX-800 spectrometers equipped with 5-mm ^13^C/^15^N/^1^H triple-resonance probes on β-PrP^23–231^ samples ranging in concentrations up to 8 mg/ml (∼330 μm). Backbone resonance assignments of β-PrP^23–231^ were achieved using the standard suite of triple resonance experiments (HNCO, HN(CA)CO, HNCACB, and CBCA(CO)NH) (40–43). Proton chemical shifts were referenced to 1 mm sodium 3-trimethylsilyl-2,2,3,3-(^2^H_4_)propionate added to the samples. ^15^N and ^13^C chemical shifts were calculated relative to sodium 3-trimethylsilyl-2,2,3,3-(^2^H_4_)propionate, using the gyromagnetic ratios of ^15^N, ^13^C, and ^1^H (^15^N/^1^H = 0.101329118, ^13^C/^1^H = 0.25144953). NMR data were processed and analyzed on Linux Workstations using Felix 2007 (Accelrys, San Diego) software. The backbone assignment was performed using the *asstools* set of assignment programs (44).

##### Analytical Ultracentrifugation

Sedimentation velocity ultracentrifugation experiments (SV-AUC) were carried out using a Beckman Optima XL-I analytical ultracentrifuge. Samples were loaded into Beckman AUC sample cells with 12-mm optical path two-channel centerpieces, with matched buffer in the reference sector. Cells were spun at 50,000 rpm in an AnTi-50 rotor, and scans were acquired using both interference and absorbance optics (at 280 nm) at 10-min intervals over 16 h. The sedimentation profiles were analyzed using the software *SEDFIT* (v13b) (45). Partial specific volumes for mouse PrP^91–231^ and PrP^23–231^ were calculated from the amino acid sequence using *SEDNTERP* software (46). Buffer densities and viscosities were measured using an Anton Paar DMA5000 density meter and an Anton Paar AMVn automated microviscometer, respectively. Sedimentation velocity data were analyzed using the *c*(*s*) method of distribution (45) to characterize the sedimentation coefficient distribution of all species present in solution. For the β-PrP samples, it was necessary to use a bimodal *f*/*f0* fit, to separately fit the frictional ratios for monomer and larger species. The proportions of each sample occupying the main peaks in the distribution were calculated by integration of the peaks.

##### Asymmetric Flow Field-Flow Fractionation

Asymmetric flow field-flow fractionation (AF4) experiments were performed using the Wyatt Eclipse 3+ with delay-corrected inline monitoring of UV (275 nm), refractive index, and light scattering coupled to an Agilent Affinity 1200 HPLC. A short channel (145 mm length) containing a 350-μm trapezoidal spacer and 5-kDa molecular mass cut-off polyethersulfone membrane was used for separation. Samples were run in 10 mm sodium acetate, 2 mm DTT, pH 4. Typically, injections of 50 μl were performed with a cross-flow of 1.5 ml/min over 1 min followed by 1 min focusing time, and elution with 1.5 ml/min cross-flow. Weight-averaged molar mass (Mw), and hydrodynamic radius were calculated, where possible, from the data using ASTRA6 software.

##### Urea Denaturation

Samples for simultaneous CD and AUC analysis were prepared in various concentrations of urea (0.0–6.0 m) in 10 mm sodium acetate, 2 mm DTT, pH 4.0, by dilution of the β-PrP^23–231^ stock solution with 9 m urea in the same buffer as appropriate. Samples were then concentrated to 50 μm PrP using Vivaspin 6 spin concentrators (Sartorius), and the flow-through used as the matched buffer reference for CD and AUC experiments.

##### Equilibrium Denaturation Data Analysis

The amide (far UV) CD absorption of 50 μm β-PrP^23–231^ in 10 mm sodium acetate, 2 mm DTT, pH 4.0, was recorded in varying concentrations of urea as described above. The mean residue ellipticity signal ([θ]r) at 215 nm was converted to the proportion of molecules in the unfolded state, α_U_, according to the relationship α_U_ = 1 − [(([θ]*r_i_* − [θ]r_5M_)/([θ]r_0M_ − [θ]r_5M_)) × 100], where [θ]r_0M_ and [θ]r_5M_ are the mean residue ellipticity values for the beginning and end of the urea denaturation transition (*i.e.* 0 and 100%, respectively). The normalized data were then fitted to a two-state unfolding equation,

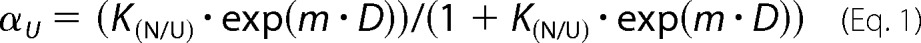
 where *m* represents the sensitivity of the unfolding transition to denaturant and *D* is the denaturant concentration (47). For the AUC data, the integrated value for percentage occupancy of monomer peak was used for α_U_ and this was used directly in the equation.

The two-state unfolding model is not formally correct for the observed β-PrP^23–231^ unfolding transition, as the data represent a transition between monomeric species at high urea concentration and oligomeric species at lower urea concentrations. The form of the CD transition does nevertheless follow that for two-state unfolding and therefore the data were fitted to the two-state unfolding model to derive minimum and maximum values for [θ]*_r_*, representing the signal of folded and unfolded species, regardless of association state. These values were then used to derive the proportion of folded species at each urea concentration.

##### Determination of Protease Resistance

α-PrP^23–231^ and β-PrP^23–231^ at pH 6.0 were digested with varying concentrations (0.005–50 μg/ml) of proteinase K (PK; British Drug Houses) at 37 °C for 1 h and diluted to 1 mg/ml in 10 mm sodium acetate, 10 mm Tris acetate, pH 6.0. Digestion was terminated by the addition of Complete ULTRA Protease Inhibitor (Roche Applied Science). Samples were heated to 100 °C for 5 min in SDS loading buffer before electrophoresis on 16% Tris glycine polyacrylamide gels (Novex). Gels were Coomassie stained.

##### Negative Stain Electron Microscopy

300-Mesh carbon-coated grids (Electron Microscopy Sciences) were glow discharged for 40 s (to make them hydrophilic) using an EMS 100× glow discharge unit (Electron Microscopy Sciences) immediately before loading the sample. 5 μl of protein at a concentration of 0.5 mg/ml was allowed to bind for 2 min, grids were blotted with grade 4 Whatman filter paper, washed twice briefly with water, then stained with 2% (*w/v*) uranyl acetate (Agar Scientific) for 45 s. Excess stain was then removed by blotting and grids were allowed to air dry. As a precaution, grids were loaded within a class 1 microbiological safety cabinet in an ACDP level III containment laboratory. Grids were analyzed using an FEI Tecnai T12 Transmission Electron Microscope (FEI, Eindhoven, NL) at an accelerating voltage of 120 kV using a dedicated sample holder for potentially infectious material and handled using appropriate safety measures. After use, the holder was decontaminated using a Plasma Cleaner (Fischione Instruments).

##### Electron Tomography

Samples were prepared in the same manner as for negative stain electron microscopy. Serial EM (48) was used to acquire a single axis tilt series with a tilt range of ± 60° with 2° increments. Digital images were recorded using a 1K 61K Gatan Multiscan 794 CCD camera at a nominal magnification of 42,000 with a pixel size of 4.1 Å and a typical defocus of 1.2 μm. IMOD version 4.3 (49) was used to reconstruct tomograms from the tilt series using local patch tracking for alignment.

##### PrP Molecular Volume Calculation

To derive estimates of the number of monomers per oligomer for the EM data for which particle dimensions were measured, two different methods to estimate the molecular volume of recombinant PrP^23–231^ were used. A molecular volume of 27.5 nm^3^ was calculated using molecular weight and the calculated partial specific volume of 0.7078 cm^3^/g. Molecular volume was also estimated using the program *Vossvolvox* (50). A modeled Protein Data Bank file of full-length PrP, comprising residues 23–231 but lacking the GPI anchor and carbohydrate moieties was used as input, giving a volume estimate of 30 nm^3^, in close agreement with the estimate using partial specific volume.

##### Cell Viability Experiments

N2a mouse neuroblastoma cells (subclone PK1) (51) chronically infected with RML prions or uninfected were cultured in OptiMEM (Invitrogen) supplemented with 10% FCS (Invitrogen) and 1% penicillin/streptomycin (Invitrogen). Cells were seeded in a 384-well plate at 6000 cells per well. Preparations of α-PrP^23–231^ at 6 mg/ml, β-PrP^23–231^ at 4.6 mg/ml, and fibrillar β-PrP^23–231^ (prepared as described above) in 10 mm sodium acetate, pH 4, and a buffer control were assessed for cytotoxicity. For each sample, a 10-fold dilution into cell media was made and then serial 3-fold dilutions were prepared. *n* = 3 wells were exposed to each concentration of PrP and buffer for 3 days prior to performing the CellTiter-Glo luminescent cell viability assay (Promega), which measures total viable cell number by quantification of ATP in whole cell lysates. Untreated cells were used as negative controls.

##### In Vivo Infectivity Experiments

30 μl of β-PrP^23–231^ at 1 mg/ml was injected into the right parietal lobes of 10 recipient CD1 mice at the age of 4–6 weeks. 10 CD1 mice were inoculated with the corresponding preparation buffer as negative controls. Mice were monitored for signs of scrapie infection throughout their normal lifetime, culled according to Home Office guidelines, and their brains taken for neuropathological examination using immunohistochemistry and Western blotting. Brain sections were checked for spongiform neurodegeneration using Harris hematoxylin and eosin staining, the proliferation of reactive astrocytes using anti-GFAP antibodies, and abnormal PrP immunoreactivity using the anti-PrP monoclonal antibody ICSM-35 (D-Gen Ltd., London) (52). Appropriate positive and negative controls were used throughout.

## RESULTS

### 

#### 

##### β-PrP^23–231^ Is Characterized by β-Sheet Structure and a Low Degree of Tertiary Organization

**FIGURE 1.**
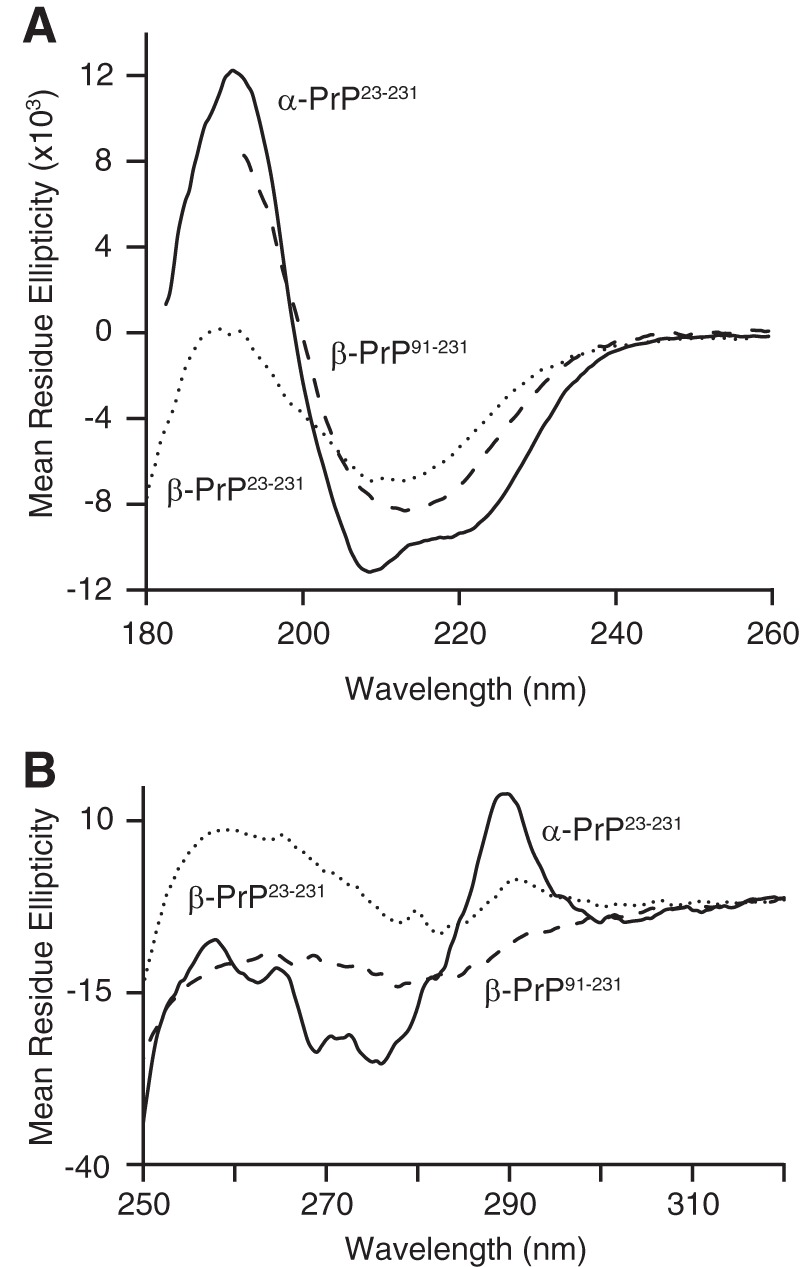
**Secondary and tertiary structure organization of α-PrP, β-PrP^91–231^, and β-PrP^23–231^.** *A*, far-UV CD spectra. Both β-PrP species (β-PrP^23–231^, *dotted line*; β-PrP^91–231^, *dashed line*) display CD spectra indicative of secondary structure dominated by β-sheet, in contrast with α-PrP^23–231^ (*solid line*), which exhibits characteristic α-helical double minima at 208 and 222 nm. *B*, near-UV CD spectra. Natively folded α-PrP displays strong near-UV CD signals (*solid line*), indicating that the side chains of its aromatic residues are in asymmetric environments characteristic of fully folded, globular proteins, and it has a well defined tertiary structure. The near-UV CD signal of both β-PrP species (β-PrP^23–231^, *dotted line*; β-PrP^91–231^, *dashed line*) are markedly reduced in comparison, consistent with a partly folded intermediate conformation lacking extensive well defined tertiary contacts. The data are representative of 6 repeats of the standard CD acquisition.

Both β-PrP^23–231^ and β-PrP^91–231^ display far-UV CD spectra characteristic of secondary structures dominated by a β-sheet, with a single minimum close to the characteristic β-sheet minimum at 215 nm (Fig. 1*A*) (53). The CD spectra are similar to those observed for a number of oligomeric and amyloid-like PrP species (54–56) and are in marked contrast to the equivalent spectra of native full-length mouse α-PrP^23–231^. The latter show a minimum at 208 nm and a shoulder at 222 nm, characteristic of a protein dominated by the α-helical secondary structure (Fig. 1*A*). The near UV (aromatic) CD spectrum of β-PrP^23–231^ displays markedly reduced intensity in comparison with that of natively folded α-PrP, indicating that the protein side chains of aromatic residues within β-PrP^23–231^ are in less asymmetric environments than in the natively folded protein (Fig. 1*B*).

##### β-PrP^23–231^ Populates a Discrete Oligomeric Species

**FIGURE 2.**
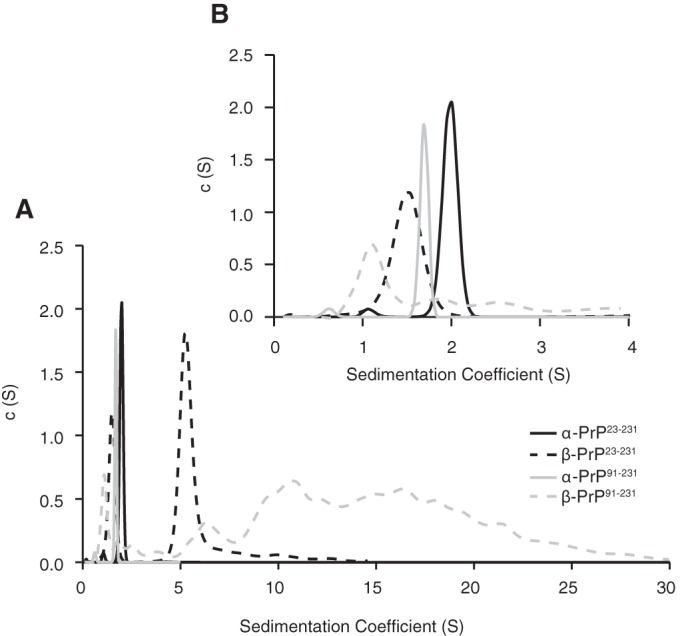
**Oligomeric states of α-PrP, β-PrP^91–231^, and β-PrP^23–231^ as assayed by SV-AUC.** Full (0–30 S) (*A*) and expanded (*B*) sedimentation coefficient distributions of α-PrP^23–231^ (*solid black line*), α-PrP^91–231^ (*solid gray line*), full-length β-PrP^23–231^ (*dashed black line*), and β-PrP^91–231^ (*dashed gray line*). Both α-PrP species sediment with derived molecular masses equivalent to the expected molecular mass of their monomers (*B*), whereas β-PrP^23–231^ is populated by a monomer and a distinct 5.6 S species, and β-PrP^91–231^ consists predominantly of a broad distribution of soluble species sedimenting between ∼1 and 30 S. For the β-PrP^23–231^ SV analysis, the average and S.D. for various parameters over 13 replicate experiments on 5 different preparations are as follows: sedimentation coefficient: monomer 1.6 ± 0.1 S, oligomer 5.6 ± 0.6 S; percentage occupancy of sample: monomer 24 ± 7%, oligomer 61 ± 13%; frictional ratio: monomer 1.8 ± 0.3, oligomer 3.0 ± 0.5.

The significant β-sheet content and reduced level of tertiary structure for β-PrP^23–231^ are features shared with β-PrP^91–231^. This truncated form of PrP was previously determined to be predominantly oligomerized under native conditions (28). We thus sought to confirm whether β-PrP^23–231^ was similarly composed primarily of oligomeric species. Intriguingly, despite displaying very similar CD spectra, the two β-PrP species, β-PrP^91–231^ and β-PrP^23–231^, were found to oligomerize in radically different manners; the former forming a broad range of soluble oligomeric species, and the latter forming a defined, distinct oligomeric state. This was shown initially using SV-AUC (Fig. 2*A*). Analysis at 1.0 mg/ml showed that β-PrP^23–231^ is comprised of two discrete species with sedimentation coefficients of 1.5 ± 0.1 and 5.6 ± 0.6 S, characterized by frictional ratios of 1.8 ± 0.3 and 3.0 ± 0.5, respectively, giving molecular masses of 23 ± 3 and 280 ± 42 kDa. These molecular masses correspond with those of the monomer and of an oligomer comprising ∼12 ± 2 monomer subunits. This is markedly different to β-PrP^91–231^, which does not populate any dominant species larger than the monomer, but rather populates a broad distribution of oligomeric species sedimenting between 2 and 30 S, with estimated molecular masses up to 2 MDa (Fig. 2*A*) (28). By way of comparison, PrP^23–231^ and PrP^91–231^ refolded into the native α-PrP conformation each show a single species sedimenting at 2 and 1.7 S, with frictional ratios of 1.6 and 1.5, respectively. These values translate to molecular masses of 23 and 17 kDa, which match closely the expected molecular masses for the monomeric species (Fig. 2*B*).

The oligomeric species in the samples (both the discrete β-PrP^23–231^ oligomer and the broad distribution β-PrP^91–231^ oligomers) are characterized by large frictional ratios *f*/*f*0 3.0 for β-PrP^23–231^ and *f*/*f*0 3.6 for β-PrP^91–231^), indicating significantly non-spherical character for both oligomers. This contrasts with the α-PrP monomers, which display lower frictional ratios, consistent with the slightly elongated structured core region (residues 121–231), connected to an unstructured N-terminal tail of 100 or 30 residues (6, 39). For both full-length and truncated PrP, the β-PrP monomers are characterized by slightly larger frictional ratios (1.8) than the α-PrP monomers, which results in their slightly slower sedimentation and smaller sedimentation coefficient (Fig. 2*B*). This is consistent with a slightly less compact monomeric state resulting from the loss of conformational restriction imposed by the native disulfide bond (7, 8).

**FIGURE 3.**
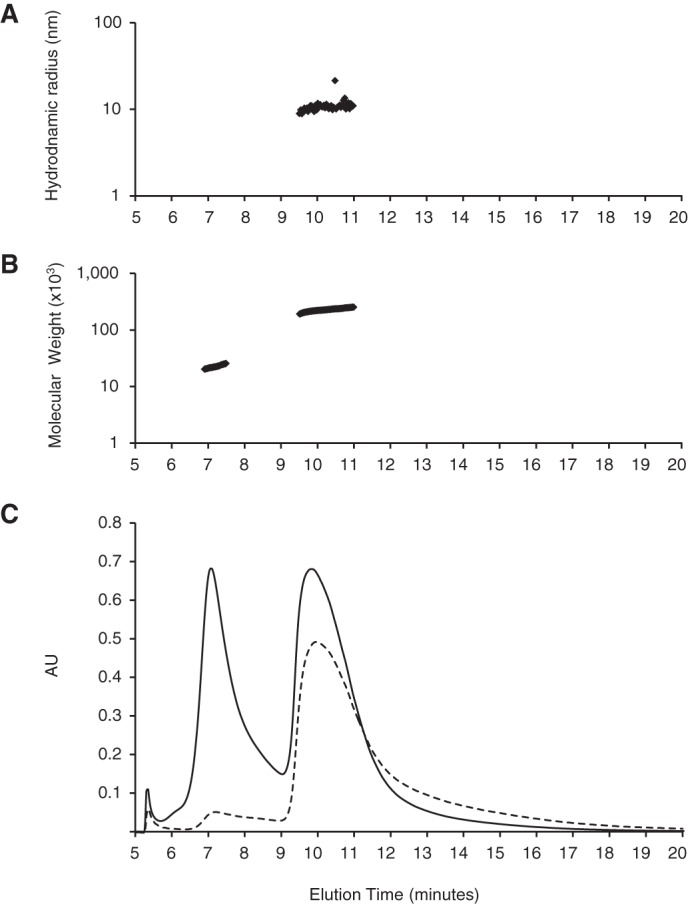
**Asymmetric-flow field-flow fractionation (AF4) of β-PrP^23–231^.** Two peaks are detected by UV (*solid line*) and light scattering (Raleigh ratio, *dashed line*) (*C*), which correspond to the monomer (elution at 7.2 min) and oligomer (elution at 10 min) species identified by AUC as shown by the derived molecular masses (23 ± 3 and 226 ± 36 kDa (*B*)). The hydrodynamic radius of the oligomers is 11 ± 2 nm as determined by dynamic light scattering (*A*). The data are representative of 10 elution runs of β-PrP^23–231^ at different concentrations from two different protein preparations.

The presence in solution of the β-PrP^23–231^ monomer and a distinct, well defined β-PrP^23–231^ oligomeric species was confirmed by asymmetric-flow field-flow fractionation (AF4), a technique that has previously been used to separate small non-fibrillar β-sheet-rich oligomers of PrP from scrapie-infected mouse brain material (12). The molecular mass determined for the oligomer using this technique was ∼226 ± 36 kDa, in agreement with the values obtained using AUC (Fig. 3), and the number of monomers in infectious prion particles (12). The monomeric species was similarly identified with a close correspondence with the AUC data (Fig. 3). The hydrodynamic radius of the oligomer was determined using in-line dynamic light scattering measurements to be ∼11 nm. Notably, the AF4 data indicated a low degree of polydispersity for the oligomeric species (1.04), indicating that the β-PrP^23–231^ oligomer was highly homogeneous.

##### β-PrP^23–231^ β-Sheet Structure Is Associated with its Oligomerization

**FIGURE 4.**
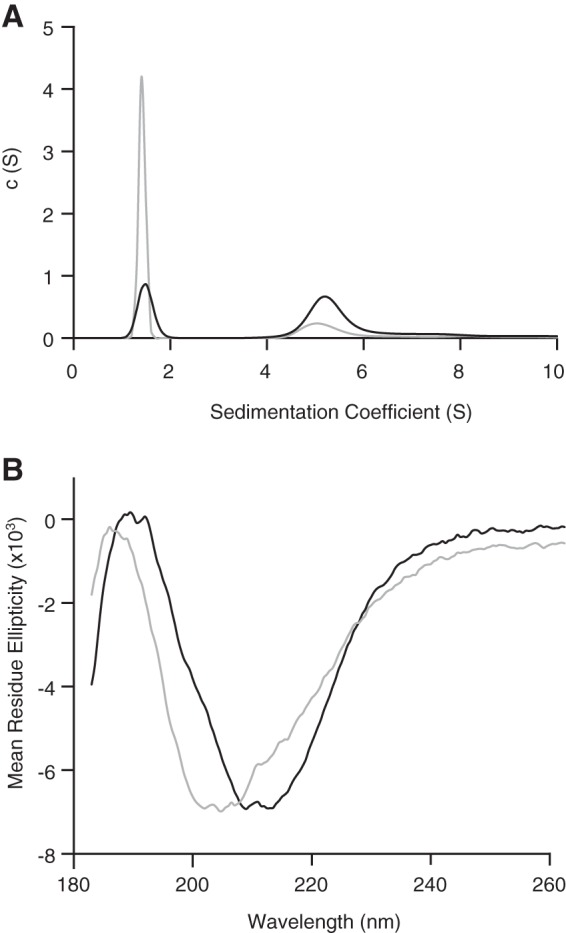
**Concentration dependence of the β-PrP^23–231^ species distribution.** β-PrP^23–231^ samples were prepared from concentrated stock (5 mg/ml; *black line*) and dilute stock (0.44 mg/ml; *gray line*), and analyzed at 0.44 mg/ml. *A*, continuous *c*(*s*) distribution of sedimentation velocity data, showing lower population of oligomer species in the sample from dilute stock compared with the concentrated stock and standard conditions (Fig. 2). *B*, the far-UV CD spectrum of the β-PrP^23–231^ diluted from concentrated stock (*black line*) displays an increased proportion of species exhibiting a β-sheet CD response (minimum at 212.5 nm), whereas the unconcentrated sample (*gray line*) contains an increased proportion of random-coil species.

**FIGURE 5.**
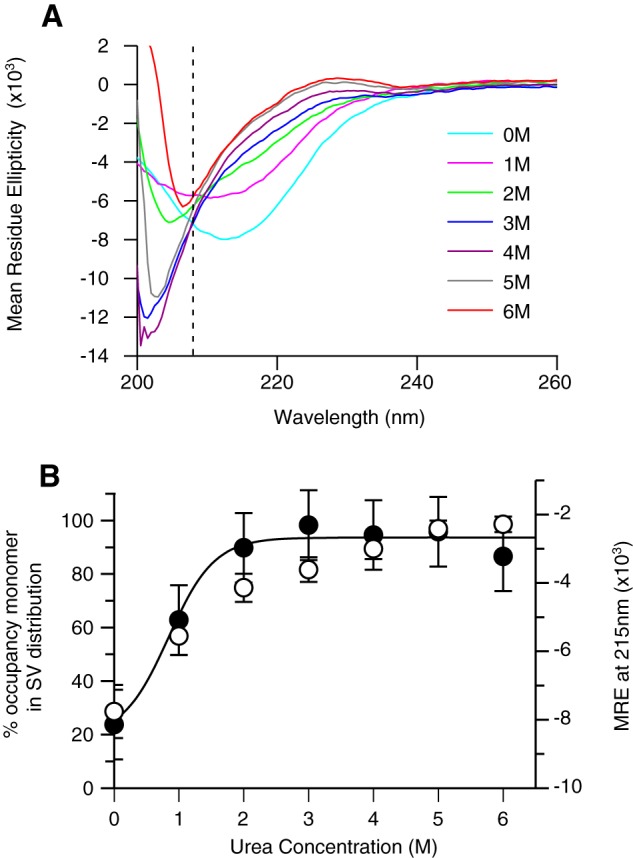
**Equilibrium denaturation of β-PrP^23–231^ monitored by far-UV CD (*A* and *B*) and SV-AUC (*B*).** *A*, far-UV CD spectra of β-PrP^23–231^ at increasing concentrations of urea, as described under “Experimental Procedures.” The isodichroic point at 206 nm is highlighted by the *dotted line*, consistent with an apparent two-state unfolding transition. *B*, the increase in percentage of monomeric species populated in the equilibrium denaturation of β-PrP^23–231^ monitored by SV-AUC (*black circles*, left-hand axis), is coincident with the loss of β-sheet CD signal (mean residue ellipticity at 215 nm, *open circles*, right-hand axis), indicating that the β-sheet secondary structure signal is lost co-incident with the dissociation into monomer. (The *solid line* shows a non-linear least squares fit of the monomer population data using a two-state model for unfolding; see “Experimental Procedures.”)

The relative proportions of the β-PrP^23–231^ monomer and oligomer could be readily changed by altering the protein concentration. The proportion of the β-PrP^23–231^ oligomeric species was found to increase from 24% to greater than 50% by elevating the β-PrP^23–231^ concentration from 0.44 to 5 mg/ml. When the protein was incubated at 5 mg/ml and then diluted back to 0.44 mg/ml this elevated oligomer/monomer ratio was retained compared with a sample refolded at 0.44 mg/ml (Fig. 4*A*), which indicates that the rate of equilibration between the oligomer and monomer is slow on the timescale of measurement (hours). Increasing the proportion of β-PrP^23–231^ oligomer was found to alter the observed far-UV CD response. The far-UV CD spectrum of a 0.44 mg/ml sample that had not been concentrated displayed a minimum at ∼208 nm, consistent with a mixed population of predominantly random coil and predominantly β-sheet conformers (Fig. 4*B*), whereas the spectrum of a 0.44 mg/ml sample that had been previously incubated at 5 mg/ml displayed a minimum at ∼213 nm indicating a shift in population in favor of β-sheet-rich conformers. This indicates that the primary β-sheet CD response is derived from the oligomers. These spectra also reflect that the monomeric form of β-PrP^23–231^ folds into a conformation that is characterized by a major loss of α-helical structure and increased proportion of unordered structure, and is qualitatively similar to the behavior of the monomeric form of β-PrP^91–231^ (57). The strong correlation between the β-sheet far-UV CD response and the aggregation state of β-PrP^23–231^ was further established through the equilibrium denaturation of β-PrP^23–231^ (Fig. 5). Comparison of the proportion of oligomeric species determined using AUC with the CD signal at 215 nm indicated that the denaturant-dependent loss of β-sheet CD signal coincided with the reduction of the oligomer population (and concomitant increase in the proportion of monomers) (Fig. 5*B*). The oligomeric species was found to unfold in a smooth transition with a low dependence on denaturant (Fig. 5*B*), indicating that the β-oligomeric species is not associated with a large scale burial of hydrophobic groups.

##### β-PrP^23–231^ Oligomer Structure

**FIGURE 6.**
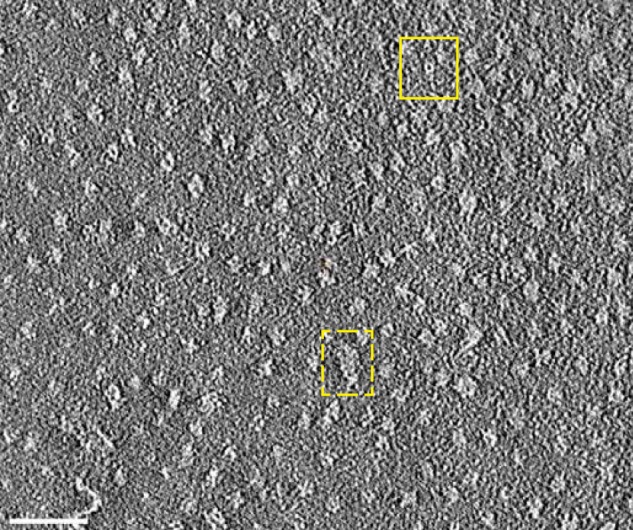
**β-PrP^23–231^ oligomers imaged by electron tomography.** A section of a tomographic reconstruction of negatively stained β-PrP^23–231^ reveals particles with dimensions of ∼8 × 9 nm, and a central cavity or cleft of ∼3 nm diameter (*solid square*), which can associate into extended structures (*dotted square*). *Scale bar*, 50 nm.

The structural organization of the β-PrP^23–231^ oligomers was characterized by electron tomography (Fig. 6). Oligomers were examined by negative stain EM and reconstructed by electron tomography. The latter reveals distinct particles with a diameter of 8.4 ± 1 nm and a height of 9 ± 1.3 nm, which can associate to form extended structures composed of 2–3 oligomers. This higher-order association of oligomers may explain the extended frictional ratios required to describe the 5 S species identified using AUC (Fig. 2). In addition, a central cavity of 2.8 ± 0.7 nm diameter (Fig. 6) is observed in many of the oligomers, similar to oligomers captured along the assembly and disassembly pathways of transthyretin amyloid protofibrils (58). The size of the particles identified lies within the size estimates expected given the molecular weight of the oligomers determined using AUC and AF4. The size estimates for the β-PrP^23–231^ oligomers (7–10 nm) and minimum assembly state (11–12 monomers) are thus in keeping with other distinct non-fibrillar PrP oligomers that have been reported (see “Discussion”).

##### NMR Characterization of β-PrP^23–231^

**FIGURE 7.**
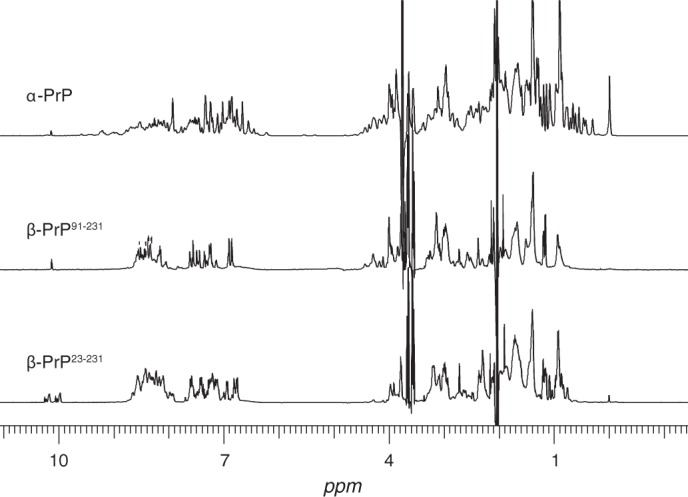
**Representative one-dimensional ^1^H NMR spectra of α-PrP, β-PrP^91–231^, and β-PrP^23–231^.** α-PrP displays a well dispersed NMR spectrum characteristic of a fully folded protein containing well defined tertiary structure. In contrast, signals observed in both β-PrP species are restricted to residues in the unstructured N terminus of the protein (up to residue 126). This is highlighted by the observed signals at ∼10.2 ppm, which arise from tryptophan side chains and occur exclusively within the unstructured N terminus. Signals from the remainder of the protein are broadened beyond detection by a combination of exchange broadening from molten globule formation and the high molecular weight soluble oligomeric species. At the protein concentration used (8 mg/ml) the dominant species in the β-PrP^23–231^ NMR sample is the oligomer.

As β-PrP^23–231^ was found to be soluble at high concentrations (∼8 mg/ml), its structural properties on a per-residue basis could be examined using solution NMR (Fig. 7), in particular allowing the determination of the involvement of the N-terminal region in the distinct oligomeric species. Intriguingly, despite oligomerizing in a radically different manner, the NMR spectrum of β-PrP^23–231^ was found to be very similar to that of the truncated β-PrP^91–231^ form. Both β-PrP species give NMR spectra displaying markedly reduced chemical shift dispersion of resonances in comparison with natively folded α-PrP. The ^1^H NMR spectrum of α-PrP (Fig. 7) is characteristic of a fully folded globular protein, with wide chemical shift dispersion, including a number of upfield-shifted methyl peaks that are found at <0.7 ppm, which are indicative of strong tertiary interactions between aromatic residues and methyl side chains. In contrast, the NMR spectra of the β-PrP species are dominated by narrow clusters of intense peaks, the majority of which lie within chemical shift ranges characteristic of random coil, unfolded peptides. As found in β-PrP^91–231^, and a number of other partially folded and oligomeric PrP states (26, 28, 55, 59), these intense peaks observed in the β-PrP^23–231^ NMR spectra were assigned to residues in the N terminus of the protein, residues 23–126. These were found to have chemical shift values very similar to those observed in the unstructured N terminus of α-PrP and β-PrP^91–231^, consistent with this region of the protein remaining predominantly unstructured in β-PrP^23–231^.

The low near-UV CD signal and lack of chemical shift dispersion in the NMR spectra are indicative of little tertiary organization within β-PrP^23–231^. However, the strong far-UV response indicates a significant β-sheet secondary structure and thus β-PrP^23–231^ behaves as a partly folded, molten globule state (60). These folding intermediates are characterized by near-native secondary structure, but little tertiary structure; the fluctuating nature of this species often results in the exchange broadening of NMR signals including their complete loss (61). Indeed, the number of observable peaks in the NMR spectra of β-PrP^23–231^ was significantly fewer than expected, indicating that there is a loss of signal through line broadening in the β-PrP^23–231^ monomer and oligomer. Thus given the lack of tertiary interactions and its oligomerized state, the majority of β-PrP^23–231^ NMR resonances appear to be broadened due a combination of molten globule intermediate exchange kinetics and the relatively long rotational correlation time of the oligomeric species. It was not possible to differentiate signals arising from the monomeric and oligomeric species.

##### β-PrP^23–231^ Is Resistant to Proteinase K Digestion

**FIGURE 8.**
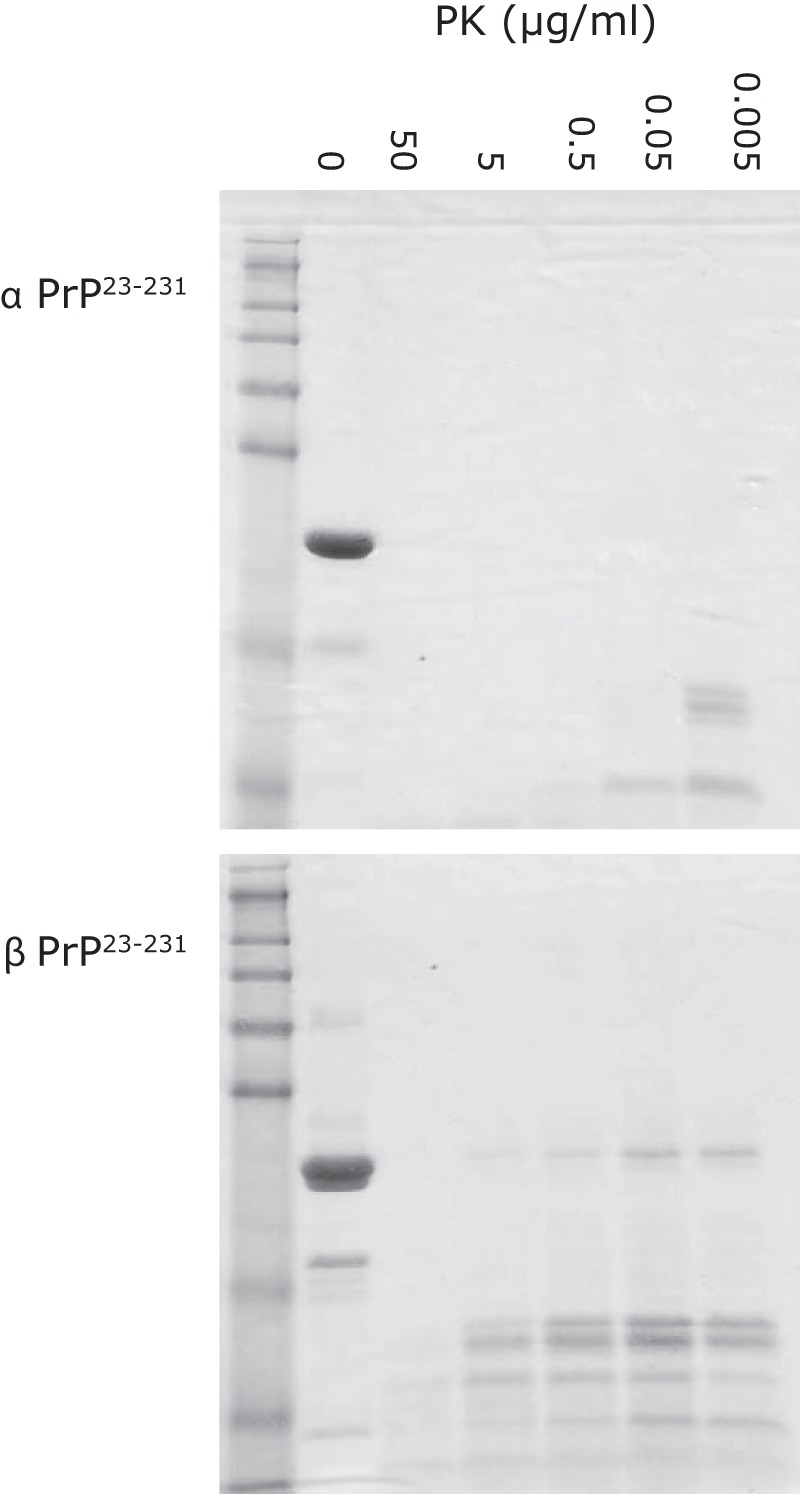
**Protease K resistance and cleavage patterns of full-length α-PrP^23–231^ (*A*) and β-PrP^23–231^ (*B*).** Protein samples were incubated at varying concentrations of proteinase K (0–50 μg/ml) in buffer at pH 6 and then analyzed by SDS-PAGE. β-PrP^23–231^ exhibited partial resistance to PK digestion, with undigested protein persisting at 5 μg/ml of PK, whereas α-PrP^23–231^ is fully digested at 0.05 μg/ml of PK. Novex SeeBlue® Plus2 Molecular weight markers are shown on the *left* of each gel.

As β-PrP^23–231^ forms such a distinct oligomeric state, rather than the broad distribution of large oligomers observed in β-PrP^91–231^, we wished to determine the relative PK resistance of β-PrP^23–231^ (Fig. 8). β-PrP^23–231^ was highly resistant to PK digestion, with undigested β-PrP^23–231^ still observed after digestion with 5 μg/ml of PK (1 h at 37 °C). The degree of protease resistance of β-PrP^23–231^ is of the same order as that observed with β-PrP^91–231^ (34), suggesting that resistance to PK digestion in both β-PrP conformers is likely to be primarily due to the structural reorganization from α-helical to β-sheet conformation rather than aggregation. In contrast, the predominantly α-helical α-PrP conformation was extremely sensitive to digestion with PK, and was completely digested by the addition of 0.05 μg/ml of PK after 1 h at 37 °C (Fig. 8).

##### β-PrP^23–231^ Forms Fibrils Under Increased Salt Concentrations

**FIGURE 9.**
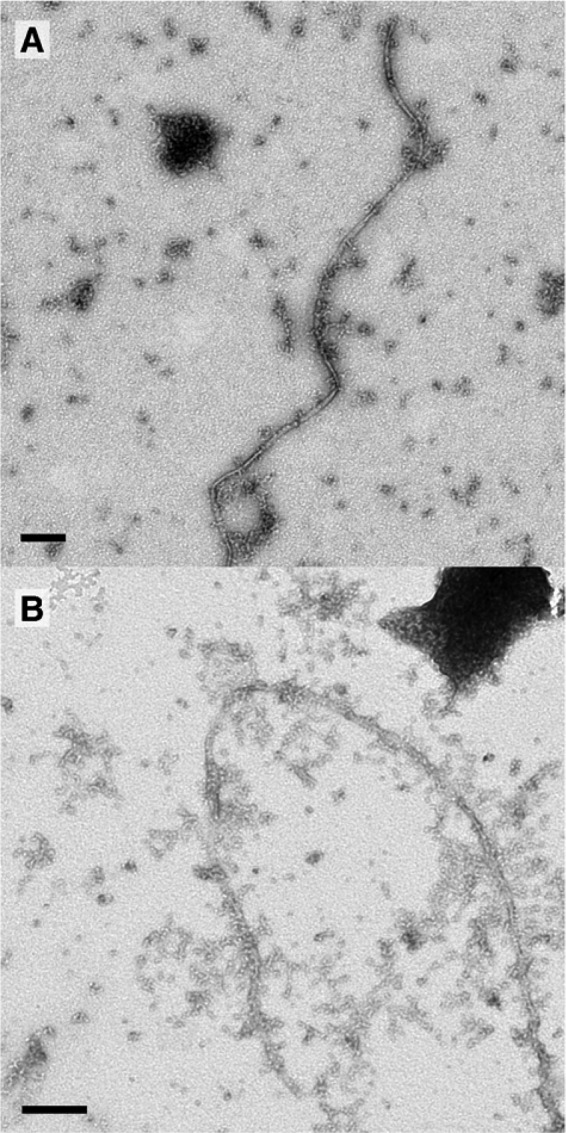
**Assembly of β-PrP^23–231^ into fibrils.** β-PrP^23–231^ is capable of associating into long unbranched fibrils, with diameters of up to 16 nm and lengths of several microns through the addition of 0.5 m GdnHCl (*A*) or 0.5 m NaCl (*B*). Both of these images are of β-PrP^23–231^ following 24 h incubation in either GdnHCl or NaCl, respectively. The *scale bar* in the images corresponds to 200 nm.

In common with β-PrP^91–231^, titration of β-PrP^23–231^ with the denaturant GdnHCl was found to increase its intermolecular association, and a conversion to oligomeric structures whose morphology could be studied by EM. This aggregation process was stimulated by equivalent concentrations of NaCl, indicating that this process is primarily an effect of increased ionic strength. Electron micrographs of β-PrP^23–231^ in 0.5 m GdnHCl and 0.5 m NaCl (Fig. 9) showed fibril-like structures with widths ranging from 10 and 16 nm, and variable lengths up to 2 μm. In addition to these, irregular amorphous aggregates of variable shape and size were also observed.

##### β-PrP^23–231^ Toxicity and Infectivity

**FIGURE 10.**
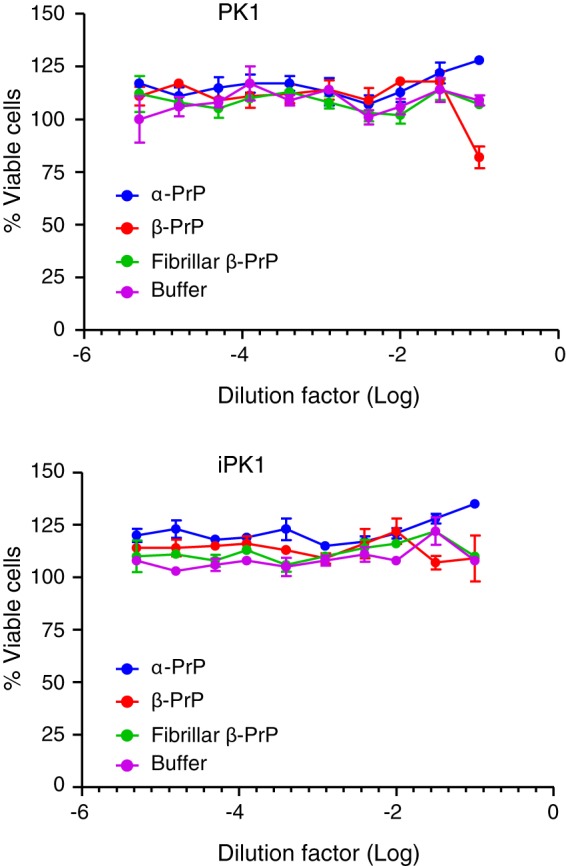
**Cell viability of uninfected (PK1) and chronically infected (iPK1) N2a cells with serial dilutions of α-PrP^23–231^, β-PrP^23–231^, and fibrillar β-PrP^23–231^ from stock solutions of 6, 4.61, and 0.25 mg/ml, respectively, with control serial dilution of buffer shown.** No significant effect on cell viability was observed from any of the three preparations of PrP or buffer used. Values were normalized to average values of untreated cells; *error bars* are S.D. expressed as percentage of the mean for *n* = *3* repeats.

We tested the effect of β-PrP^23–231^ on the viability of neuroblastoma cells (PK1) (51) by serial dilutions of the preparation into the cell growth media. No significant effect on cell viability was observed using an oligomeric β-PrP preparation at concentrations ranging from 0.002 and 460 μg/ml (Fig. 10). Fibrillar preparations of β-PrP^23–231^, and natively folded α-PrP^23–231^ over similar concentration ranges also did not affect the PK1 cell viability. This cell system thus provides no evidence for a toxic effect mediated by the β-PrP oligomers when applied externally.

The infectivity of β-PrP^23–231^ was also investigated. 10 CD1 mice were observed for 600 days (close to natural lifespan) for development of prion disease pathology following intracerebral injection of β-PrP^23–231^ (into the right parietal lobe). 10 control CD1 mice were inoculated with the corresponding β-PrP^23–231^ preparation buffer. No mice developed clinical signs of murine scrapie. The brains of all mice were removed for neuropathological examination and Western blot analysis at the end of the experiment to determine whether there was evidence of subclinical prion infection. There was no prion neuropathology on histological or immunocytochemical analysis and no PrP^Sc^ was detected by Western blot analysis.

## DISCUSSION

The molecular basis of prion neurotoxicity remains largely unknown. Loss of PrP^C^ function, as it is sequestered into aggregated forms, is not a sufficient cause because its depletion does not result in neurodegeneration (62, 63). It is increasingly recognized that multiple disease-related forms of PrP, other than PrP^Sc^, exist and may differentially contribute to prion infectivity or toxicity, which can be uncoupled (64). Interest has focused on the role of small non-fibrillar β-sheet-rich oligomers in prion and other amyloid diseases (12, 14, 16–19, 21, 32). In particular, it has been proposed that soluble oligomers are common to most amyloidotic diseases and may represent the primary toxic species (65). The distinct oligomer formed by the reduced and acidified form of full-length recombinant PrP is thus of interest as a candidate toxic or infectious species.

Here, we show that full-length recombinant prion protein, comprising residues 23–231, can be refolded *in vitro* into a well defined non-fibrillar β-sheet-rich oligomeric form. These oligomers, formed in the folding of the full-length protein contrast markedly with similar reduction and refolding of truncated PrP residues 91–231. Our study provides a detailed structural characterization of this previously uncharacterized PrP oligomeric structure.

Electron tomograms show particles with dimensions ∼8 × 9 nm, often containing a cavity or cleft. Using the monomer molecular volume (see “Experimental Procedures”), and the calculated volume of the TM particles (assuming an annular structure), the number of PrP^23–231^ molecules per oligomer is estimated to be 16 ± 5. Given the assumption that the entire volume of the particle is occupied by PrP molecules, the true oligomer number is likely to be smaller, making this value broadly consistent with the molecular mass estimates determined by the solution methods AF4 and AUC, which indicate ∼12 PrP^23–231^ molecules per oligomer (∼280 kDa). These solution methods have a major advantage over size exclusion chromatography, typically used to characterize oligomeric species and protein association states, in that they can be used to analyze samples at very low ionic strength. The structure of the oligomer is similar to that identified for oligomers captured along the assembly and disassembly pathways of transthyretin amyloid protofibrils (58).

Both the distinct β-PrP^23–231^ oligomer and the aggregated species formed by β-PrP^91–231^ share similar far-UV CD spectra indicating a dominance of β-sheet structure. The formation of β-sheet structure for both proteins is concomitant with their oligomerization; the monomeric states exhibiting little defined secondary structure and therefore relatively silent by CD spectroscopy. For both CD and NMR the size of the species cannot be determined from their respective signals, and neither can the monomer and oligomer be differentiated. The complementary solution methods used here have proved necessary to enable the protein association state to be verified independently of the spectroscopic signal. The volume and size measurements indicated by these different techniques are similar to a number of other discrete non-fibrillar oligomers formed from recombinant PrP under various *in vitro* folding conditions (13, 21, 23–27, 29–32, 66).

Although β-PrP is recognized differentially by the host immune system (36) and antibodies raised against it immunoprecipitate disease-associated PrP^Sc^ (but not native PrP^C^) the oligomeric preparation used in this study was not found be infectious, nor to affect PK1 cell viability, when applied externally.

The oligomers characterized here are broadly similar in oligomeric size and β-sheet structure to those identified as the most infectious particles from scrapie-infected mouse brain material (12). However, the generation of high titer prions from recombinant PrP remains contentious (64, 67, 68), with studies often indicating a requirement of polyanions and/or other brain-derived material for infectivity (68, 69). Confirmation of whether β-PrP^23–231^ requires interaction with an as yet unidentified co-factor for infectivity remains to be determined. It has been proposed that PrP-lipid charge interactions are required as a first step in the conversion of PrP^C^ to PrP^Sc^, consistent with this conformational transition occurring in lipid raft domains (70, 71), and membrane-like conditions are involved in the formation of a similarly sized oligomeric PrP intermediate (21, 32). The precise subcellular localization of PrP^Sc^ propagation, however, remains ill-defined, and late endosome-like organelles or lysosomes with acidic pH are, however, also implicated (72, 73).

Although we do not observe a specific cellular toxicity of this β-PrP^23–231^ preparation, several recombinant β-oligomers are reported to be toxic to neuronal cell cultures (13, 29, 30, 66, 74, 75). The variety of cell types tested, the various preparations of recombinant PrP oligomers, and the considerable experimental variation may explain variability in toxicity, but it is also probable that subtle differences between the various species under investigation can result in large differences in biological effect. Indeed this has been reported for the bacterial protein HypF-N; this protein forms two oligomers that are similar according to AFM and thioflavin T reactivity, but toxicity is displayed by the one that is characterized by a lower degree of hydrophobic packing (76). The reducing/acidic conditions for the β-PrP oligomerization may be unfavorable for the production of toxic/infectious oligomers, however, it is also possible that β-PrP toxicity may occur via a more discrete mechanism, or biological activity requiring perhaps other specific binding partners (for example, the PrP/Aβ interaction) (77), in which only certain transient Aβ assemblies cause PrP-dependent toxicity (20), and thus may require the application of the oligomer to more specialized models.

Notably, formation of this distinct oligomer requires residues 23–90. This region intriguingly remains unstructured and not directly incorporated into the β-sheet-rich core of the oligomer. There is precedence for the unstructured N-terminal region of PrP affecting the folding and distribution of oligomeric species formed by the remainder of the protein. For example, residues 105–120 are required for the conversion of α-PrP into a soluble β-sheet-rich oligomeric species (70). Indeed, intrinsically disordered domains have been shown to be involved in protein-protein interactions (78), and there is also evidence that the interaction of PrP^C^ with β-sheet-rich conformers, and the induction of the pro-apoptotic signaling is dependent on the intrinsically disordered N-terminal region of PrP^C^ (79).

Given that the N terminus of PrP contains a considerable number of charged residues (pI PrP^91–231^ = 7.95; pI PrP^23–231^ = 9.39), this suggests a principal role for electrostatic interactions in the formation of the distinct oligomeric state of β-PrP^23–231^. The highly charged N terminus of the protein may be involved in directing the formation of distinct oligomeric states that are not readily accessible to the truncated molecule as residues within this region transiently contact the folded region of PrP (6, 80, 81). Alternatively, charged surfaces in plasticware or the membrane surfaces used in dialysis may be involved, but of course this could also happen *in vivo* where there are a multitude of charged surfaces available to direct the assembly of discrete oligomeric species. For example, association of negatively charged lipids or detergents with PrP are required for its efficient conversion to a β-sheet-rich oligomeric form (32, 70). A role for the involvement of catalytic surfaces directing the formation of distinct assembly states thus remains a distinct possibility.

In conclusion, we have characterized a novel oligomeric PrP structure, the formation of which requires the full prion polypeptide chain. This highlights that to examine the full repertoire of conformers and assembly states that can be accessed by prion protein under specific experimental conditions ideally full-length prion protein should be used.

## Footnotes

8 The abbreviations used are:

PrP: prion protein
SV-AUC: sedimentation velocity analytical ultracentrifugation
PrP^C^: cellular PrP isoform
PrP^Sc^: pathogenic (scrapie) PrP isoform
PrP^23–231^: prion protein residues 23–231
PrP^91–231^: prion protein residues 91–231
GdnHCl: guanidine hydrochloride
PK: proteinase K
BisTris: 2-[bis(2-hydroxyethyl)amino]-2-(hydroxymethyl)propane-1,3-diol.
